# Supplementary material for: Intercellular communication atlas reveals Oprm1 as a neuroprotective factor for retinal ganglion cells
Source: Nat Commun. 2024 Mar 11;15:2206. doi: 10.1038/s41467-024-46428-z (PMC11636819; doi:10.1038/s41467-024-46428-z)
Supplement: Supplementary file 1 — Supplementary Information [file 41467_2024_46428_MOESM1_ESM.pdf]

Intercellular communication atlas reveals Oprm1 as a neuroprotective factor for retinal ganglion cells

Cheng Qian[1,#], Ying Xin[2,#], Cheng Qi[1], Hui Wang[3], Bryan C. Dong[4], Donald J. Zack[2], Seth Blackshaw[5,2], Samer Hattar[3], Feng-Quan Zhou[1,5,6,*], Jiang Qian[2,*]

[1] Department of Orthopaedic Surgery, Johns Hopkins University School of Medicine, Baltimore, United States.
[2] Department of Ophthalmology, Johns Hopkins University School of Medicine, Baltimore, United States.
[3] Section on Light and Circadian Rhythms, National Institute of Mental Health, Bethesda, Maryland, United States
[4] Neuroscience Study Program, Krieger School of Arts & Sciences, Johns Hopkins University, Baltimore, United States.
[5] Solomon H. Snyder Department of Neuroscience, Johns Hopkins University School of Medicine, Baltimore, United States.
[6] Current address: Sir Run Run Shaw Hospital, Zhejiang University School of Medicine, Hangzhou, China

Correspondence and requests for materials should be addressed to F.Z. or J.Q.
Feng-Quan Zhou: fzhou4@zju.edu.cn
Jiang Qian: jiang.qian@jhmi.edu

Supplementary Table 1. Numbers of retinal cells at four conditions.

	Ctrl	12h	1d	2d
Rods	5777	2502	5258	4051
coneBC	2840	1908	3583	3339
RPE	373	75	302	319
RodBC	1817	987	1871	1916
GlyAC	493	359	823	906
MG	1812	1174	2098	1927
Cones	1639	519	1551	1444
HC	386	113	368	478
GABAAC	169	168	345	331
Microglia	106	68	75	115
Pericytes	42	25	35	37
Astrocytes	97	53	108	82
VE	164	83	180	185
RGC	37	179	303	536

Supplementary Table 2. Predicted neuroprotective interactions. The sender cells are listed in the first line, and the receiver RGC subclasses are in the table content.

	Astrocytes	*Pericytes*	*GABAAC*	*GlyAC*	*HC*	*MG*	*VE*	*ConeBC*	*Cones*	*RodBC*	*Rods*	*RPE*	*Microglia*
Ncam1_Robo1	a;ip;Gpr88	a;ip;Gpr88	a;ip;Gpr88	a;Gpr88	a;ip;Gpr88	a;ip;Gpr88	a;Gpr88	a;ip;Gpr88	a;Gpr88	a;ip;Gpr88	a;ip;Gpr88	a;Gpr88	a;Gpr88
Tgfb2_Tgfbr2	a;ip;Gpr88	a;ip;Gpr88		a;ip;Gpr88	a;ip;Gpr88	a;ip;Gpr88	a;ip;Gpr88	a;ip;Gpr88		a;ip;Gpr88		a;Gpr88	
Tgfb2_Tgfbr3	a;ip;Gpr88	a;ip;Gpr88		a;ip;Gpr88	a;ip;Gpr88	a;ip;Gpr88	a;ip;Gpr88	a;ip;Gpr88		a;ip;Gpr88			
Cadm1_Nectin3	a;ip;Gpr88	a	a;ip;Gpr88	a;ip;Gpr88	a	a;ip;Gpr88	a;ip;Gpr88	a;ip;Gpr88	a;ip;Gpr88	a;ip;Gpr88	a;ip;Gpr88	a	a;ip;Gpr88
Ltbp1_Itgb5	a;ip;Gpr88	a;ip;Gpr88											
Mdk_Itga6	a;ip;Gpr88					ip;Gpr88						a;ip;Gpr88	
Ltbp3_Itgb5	a;ip;Gpr88											a;Gpr88	
Col2a1_Itga3	a;ip;Gpr88												
Ncam1_Gfra1	a;Gpr88	a;ip;Gpr88	a;ip;Gpr88	a;ip;Gpr88	a;ip;Gpr88	a;ip;Gpr88	a;Gpr88	a;ip;Gpr88	a;ip;Gpr88	a;ip;Gpr88	a;ip;Gpr88	a;Gpr88	a;Gpr88
Cadm1_Crtam	a;Gpr88	Gpr88	a;Gpr88	a;Gpr88	a;Gpr88	a;ip;Gpr88	a;Gpr88	a;Gpr88	a;Gpr88	a;Gpr88	a;Gpr88	Gpr88	a;Gpr88
Vim_Cd44	a;Gpr88	a;Gpr88				a;Gpr88	a;Gpr88						
Fn1_Cd44	a;Gpr88						a;Gpr88						
Vcan_Cd44	a;Gpr88												
Col1a2_Cd44	a;Gpr88												
Robo1_Robo1	a;Gpr88												
Bdnf_Ngfr	a;ip		a;ip	a;ip		a;ip		a;ip		a;ip;Gpr88		a;ip	
Pdyn_Oprm1	a;ip												
Penk_Oprm1	a;ip												
Tgfb1_Tgfbr2	a	a;ip;Gpr88	a;Gpr88				a;ip;Gpr88						a;ip;Gpr88
Afdn_Nectin3	a	a	a	a	a	a	a	a		a	a		
Nectin3_Nectin3	a					a				a			
Fn1_Plaur	a						a						
Anxa1_Dysf	a					a;Gpr88							
Cadm3_Nectin3			a	a	a;ip			a	a				
Nectin1_Nectin3			a		a			a	a	a	a	a	
Lamb1_Itgb1		a;ip;Gpr88	a;ip;Gpr88										
Tgfb1_Tgfbr3		a;ip;Gpr88	a;ip				a;ip;Gpr88						a;ip;Gpr88
Vtn_Itgb5		a;ip;Gpr88				a;Gpr88	a;ip;Gpr88					a;ip;Gpr88	
Thbs1_Itga6		a;ip;Gpr88										a;ip;Gpr88	
Lama2_Itga7		a;ip;Gpr88											
Lamb1_Itga6		ip;Gpr88	Gpr88				ip;Gpr88					ip;Gpr88	
Ngf_Ngfr		a;ip											
Vtn_Plaur		a					a						
Ntf3_Ngfr		a											
Tgfb3_Tgfbr3		a											
Lamc3_Itga7		ip											
Col3a1_Ddr2		Gpr88											
Adam9_Itga6		Gpr88									ip;Gpr88	a	
Lamc3_Itga6		Gpr88											
Hbegf_Cd44			a;Gpr88										
Tnc_Itga7			a;ip										
Fgf1_Cd44					a;Gpr88								
Lgals1_Itgb1						a;ip;Gpr88							
Inhba_Tgfbr3						a;ip;Gpr88							
Slit2_Robo1								a;Gpr88					
Thbs1_Itgb1												a;ip;Gpr88	
Gpc3_Igf1r												a;ip;Gpr88	

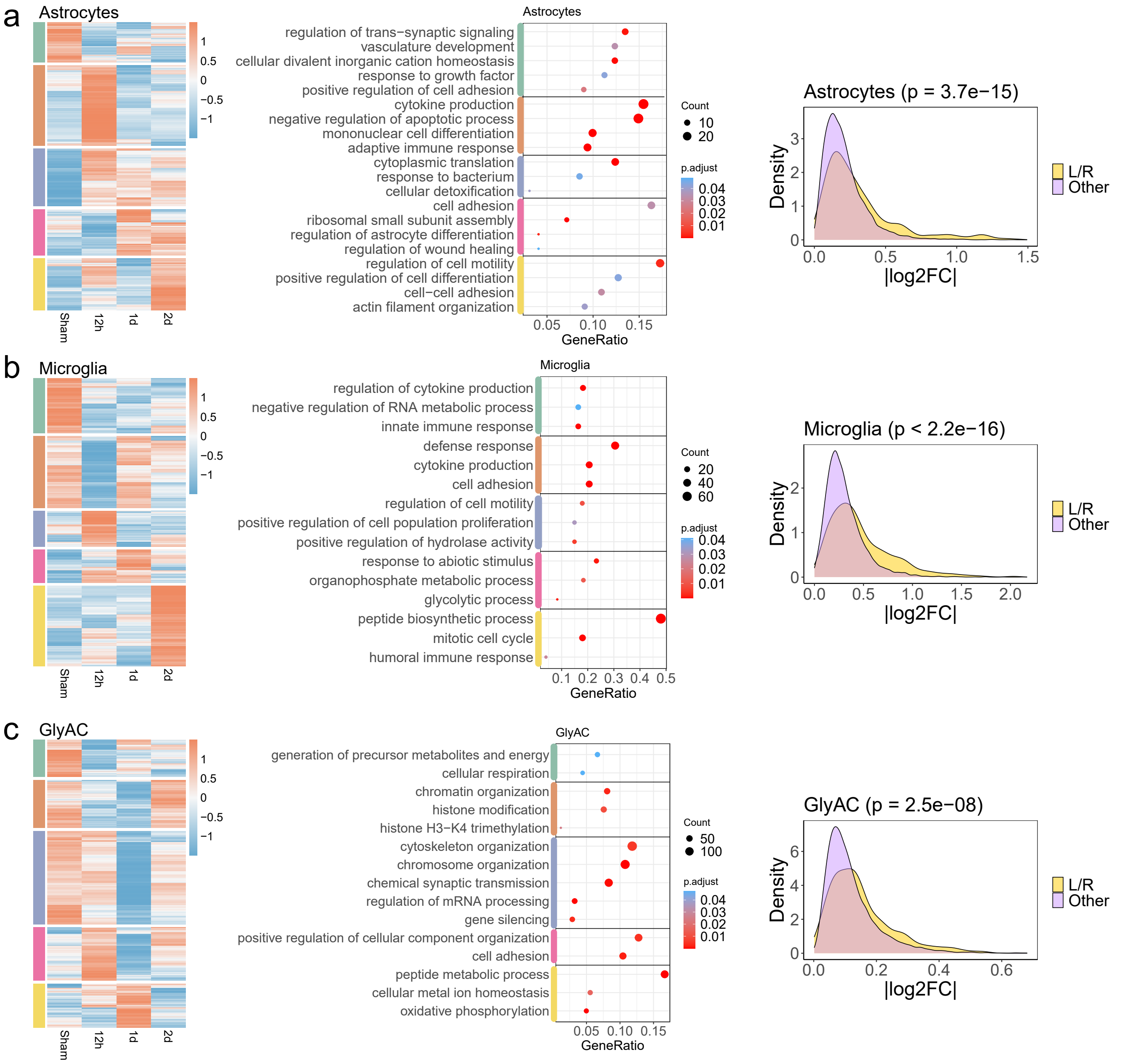

a
Astrocytes
1
0.5
0
−0.5
−1
Sham
12h
1d
2d
Astrocytes
regulation of trans−synaptic signaling
vasculature development
cellular divalent inorganic cation homeostasis
response to growth factor
positive regulation of cell adhesion
cytokine production
negative regulation of apoptotic process
mononuclear cell differentiation
adaptive immune response
cytoplasmic translation
response to bacterium
cellular detoxification
cell adhesion
ribosomal small subunit assembly
regulation of astrocyte differentiation
regulation of wound healing
regulation of cell motility
positive regulation of cell differentiation
cell−cell adhesion
actin filament organization
0.05 0.10 0.15
GeneRatio
Count
10
20
p.adjust
0.04
0.03
0.02
0.01
Astrocytes (p = 3.7e−15)
Density
|log2FC|
L/R
Other
b
Microglia
Microglia
regulation of cytokine production
negative regulation of RNA metabolic process
innate immune response
defense response
cytokine production
cell adhesion
regulation of cell motility
positive regulation of cell population proliferation
positive regulation of hydrolase activity
response to abiotic stimulus
organophosphate metabolic process
glycolytic process
peptide biosynthetic process
mitotic cell cycle
humoral immune response
0.1 0.2 0.3 0.4 0.5
GeneRatio
Count
20
40
60
Microglia (p < 2.2e−16)
c
GlyAC
GlyAC
generation of precursor metabolites and energy
cellular respiration
chromatin organization
histone modification
histone H3−K4 trimethylation
cytoskeleton organization
chromosome organization
chemical synaptic transmission
regulation of mRNA processing
gene silencing
positive regulation of cellular component organization
cell adhesion
peptide metabolic process
cellular metal ion homeostasis
oxidative phosphorylation
0.05 0.10 0.15
GeneRatio
Count
50
100
GlyAC (p = 2.5e−08)

Fig. S1 Retinal cells in response to ONC.
Response in astrocytes (**a**), microglia (**b**), and glycinergic amacrine cells (**c**), as some examples, after the ONC injury on RGCs. Left panels: DEG heatmaps show dynamical patterns of DEGs at different time points after ONC. Middle panels: Gene ontology analysis reveals representative biological processes enriched by the DEGs in each pattern shown in the heatmaps. Right panel: Density plots of the absolute fold-change (log2FC) values of genes expressed (detection rate > 0.1). Ligand and receptor genes (L/R) are grouped in yellow, while the other genes are shown in light purple for comparison. For the GO analysis in the middle panels, the p-values are based on a hypergeometric test, adjusted by the Benjamini-Hochberg method. For all right panels, the p-values are based on Kolmogorov-Smirnov test (two-sided).

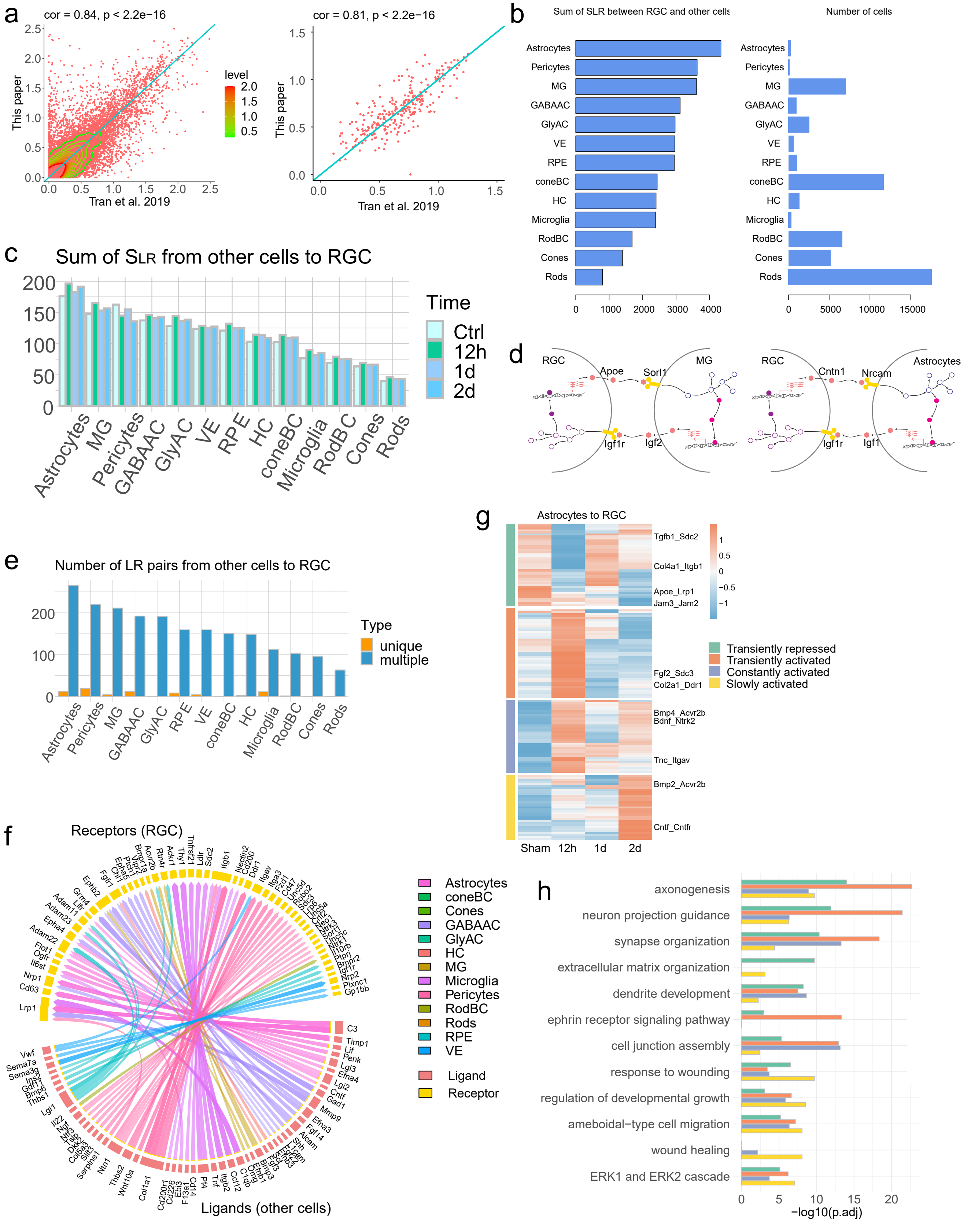

a
cor = 0.84, p < 2.2e−16
cor = 0.81, p < 2.2e−16
This paper
Tran et al. 2019
level
b
Sum of SLR between RGC and other cells
Number of cells
Astrocytes
Pericytes
MG
GABAAC
GlyAC
VE
RPE
coneBC
HC
Microglia
RodBC
Cones
Rods
c
Sum of SLR from other cells to RGC
Time
Ctrl
12h
1d
2d
d
RGC
Apoe
Sorl1
MG
Igf1r
Igf2
Cntn1
Nrcam
Astrocytes
Igf1
e
Number of LR pairs from other cells to RGC
Type
unique
multiple
f
Receptors (RGC)
Ligands (other cells)
Ligand
Receptor
g
Astrocytes to RGC
Tgfb1_Sdc2
Col4a1_Itgb1
Apoe_Lrp1
Jam3_Jam2
Fgf2_Sdc3
Col2a1_Ddr1
Bmp4_Acvr2b
Bdnf_Ntrk2
Tnc_Itgav
Bmp2_Acvr2b
Cntf_Cntfr
Sham
Transiently repressed
Transiently activated
Constantly activated
Slowly activated
h
axonogenesis
neuron projection guidance
synapse organization
extracellular matrix organization
dendrite development
ephrin receptor signaling pathway
cell junction assembly
response to wounding
regulation of developmental growth
ameboidal−type cell migration
wound healing
ERK1 and ERK2 cascade
−log10(p.adj)

Fig. S2 Responsive interactions from other cells to RGCs.
a Correlation of gene expression between RGCs captured and sequenced in whole retina data in this paper and the RGCs in Tran. et al. Pearson's correlation coefficient: p-value calculated from t-test. **b** Sum of interaction strength and the number of cells for the retinal cell types. There is no correlation between the interactions detected and cell counts. **c** Sum of the interactions at different time points for each cell type. **d** Additional examples of cell-cell feedback loops. The ligand from the sender cell interacts with the receptor on the receiver cell, triggering gene transcription in receiver cells, in which some ligand genes are transcribed and sent back to the original sender cells. **e** The number of ligand-receptor interactions from other retinal cells to RGCs. Interactions identified in multiple and unique cell types are dark blue and orange. **f** The specific ligand-receptor interactions identified in unique retinal cell types to RGCs are shown in orange columns in panel **e**. Genes in the bottom half of the circle are the ligands secreted from other retinal cells, and the genes in the top half of the circle are the receptors in RGCs. The colors of the connecting edges represent the receiver cell types. **g** Heatmap reveals the ONC-induced temporal patterns of interactions from astrocytes to RGCs across time points (the fold change (FC) of the interaction scores between any two time points > 1.2). Four dynamical patterns were identified. **h** Gene Ontology analysis (GO) reveals representative biological processes of variable ligand-receptor interactions in patterns identified in panel **g**. The enrichment was calculated with all expressed genes in either astrocyte or RGC at any time point as the background (detection rate > 0.1). The p-values are based on a hypergeometric test, adjusted by the Benjamini-Hochberg method.

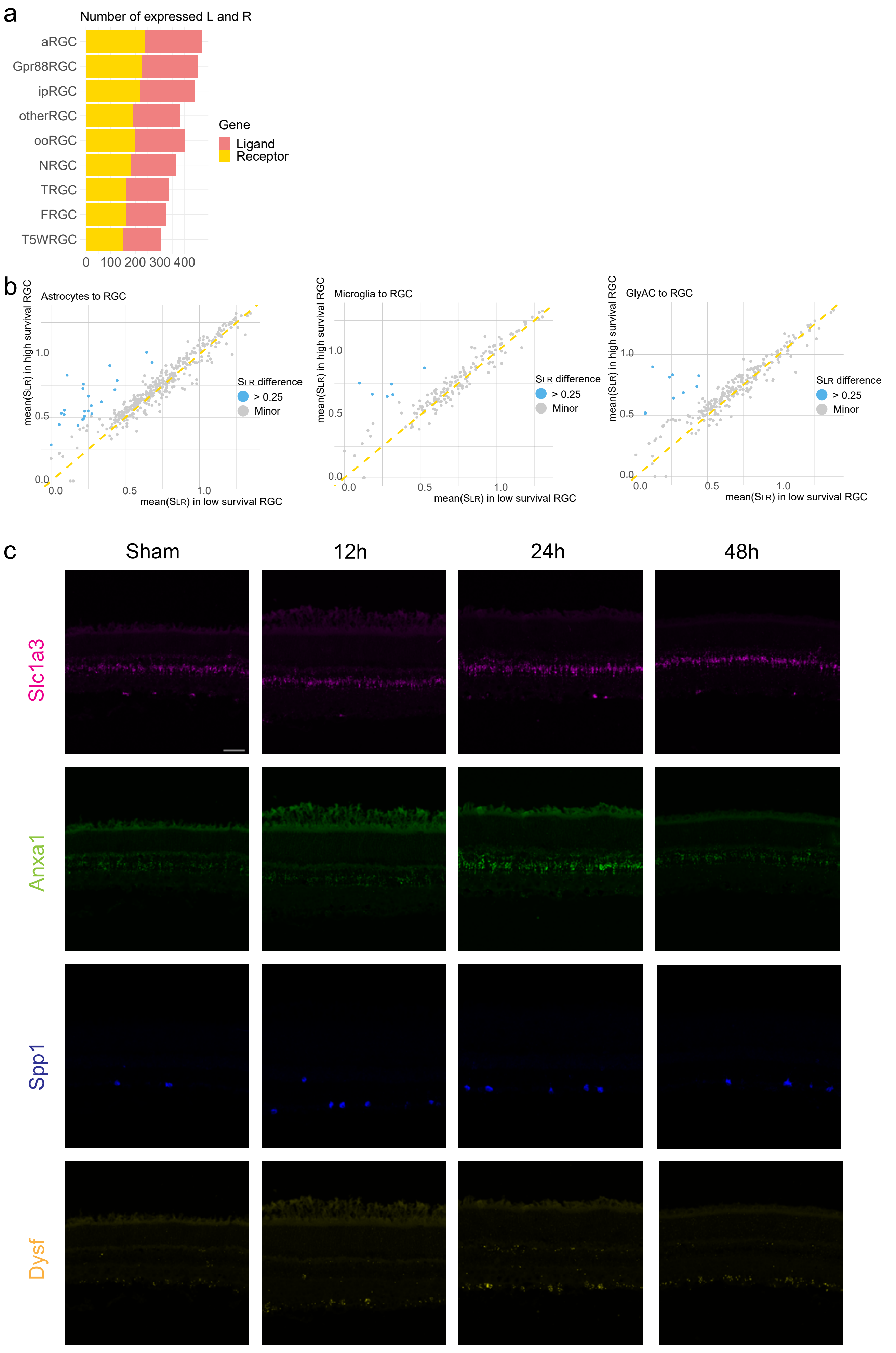
a
Number of expressed L and R
aRGC
Gpr88RGC
ipRGC
otherRGC
ooRGC
NRGC
TRGC
FRGC
T5WRGC
0 100 200 300 400
Gene
Ligand
Receptor
b
Astrocytes to RGC
Microglia to RGC
GlyAC to RGC
mean(SLR) in high survival RGC
mean(SLR) in low survival RGC
SLR difference
> 0.25
Minor
0.0
0.5
1.0
c
Sham
12h
24h
48h
Slc1a3
Anxa1
Spp1
Dysf

Fig. S3 Properties of high-survival vs low-survival RGC subclasses.

a Number of expressed ligand and receptor genes in RGC subclasses (detection rate > 0.1 at any time point). **b** Additional examples for calculating protective interactions in astrocytes (left graph), microglia (middle graph), and glycinergic amacrine cells (right graph). For each ligand-receptor pair, the two calculated mean values of interaction scores (y-axis) for the high- and the other (x-axis) for the low-survival RGC subclasses were plotted for comparison. Blue dots are the interactions that are stronger in high- than in low-survival RGC subclasses. Gray dots are the interactions with similar interaction scores between the two categories. **c** The single-channel images for **Fig. 3d**. Scale bar: 50 μm.

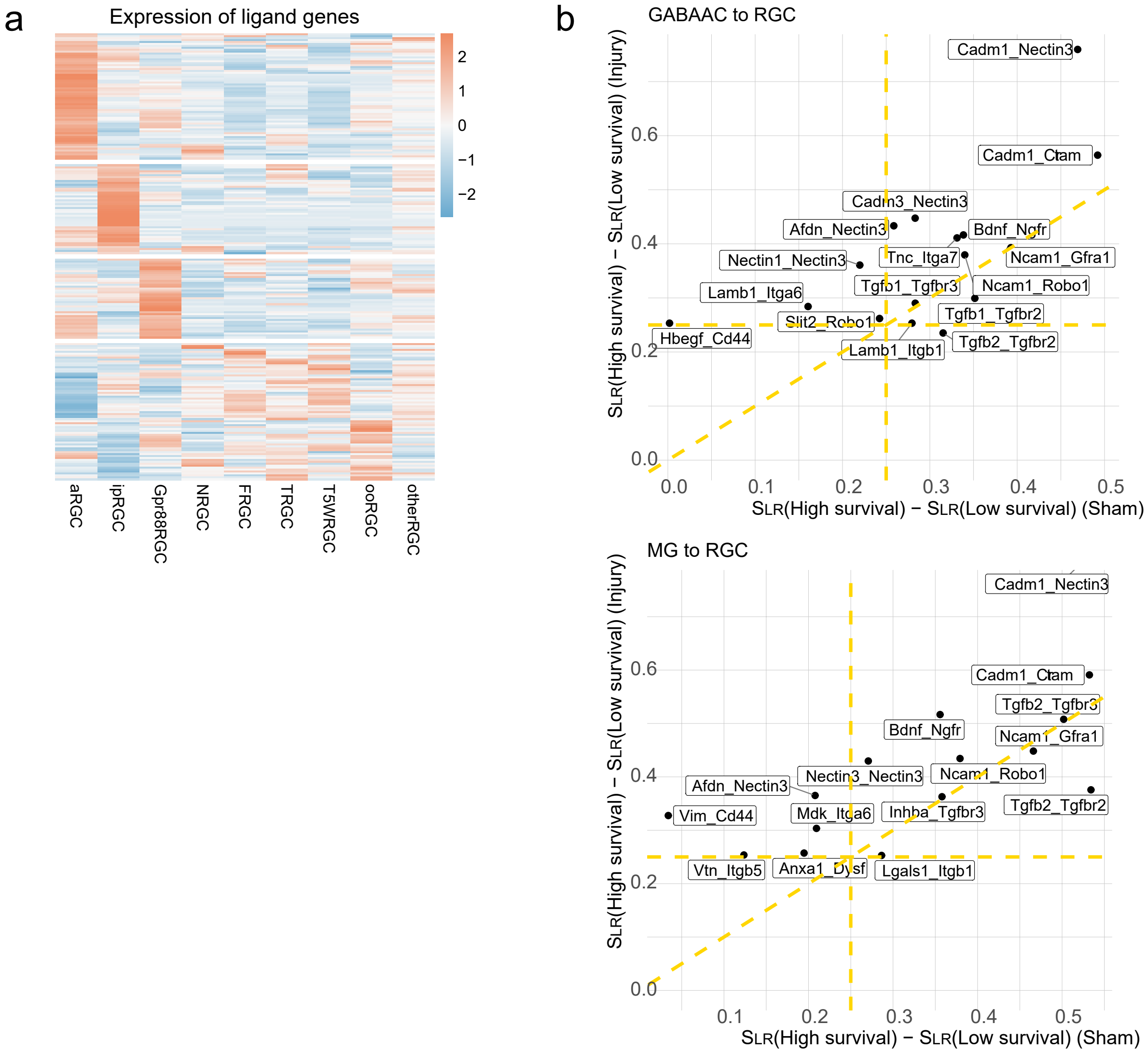
a
Expression of ligand genes
2
1
0
−1
−2
aRGC
ipRGC
Gpr88RGC
NRGC
FRGC
TRGC
T5WRGC
ooRGC
otherRGC
b
GABAAC to RGC
SLR(High survival) − SLR(Low survival) (Injury)
SLR(High survival) − SLR(Low survival) (Sham)
0.0
0.2
0.4
0.6
0.1
0.3
0.5
Cadm1_Nectin3
Cadm1_Crtam
Cadm3_Nectin3
Afdn_Nectin3
Bdnf_Ngfr
Nectin1_Nectin3
Tnc_Itga7
Ncam1_Gfra1
Lamb1_Itga6
Tgfb1_Tgfbr3
Ncam1_Robo1
Slit2_Robo1
Tgfb1_Tgfbr2
Hbegf_Cd44
Tgfb2_Tgfbr2
Lamb1_Itgb1
MG to RGC
Cadm1_Nectin3
Cadm1_Crtam
Tgfb2_Tgfbr3
Bdnf_Ngfr
Ncam1_Gfra1
Nectin3_Nectin3
Ncam1_Robo1
Afdn_Nectin3
Mdk_Itga6
Inhba_Tgfbr3
Tgfb2_Tgfbr2
Vim_Cd44
Vtn_Itgb5
Anxa1_Dysf
Lgals1_Itgb1

Fig. S4 Features of protective interactions.
a Average expression level of ligand genes in RGC subclasses. **b** Summary of the preset and induced protective interactions. The X-axis is the difference between interaction scores in high- and low-survival subclasses before injury, and the Y-axis is the interaction score difference after injury. Interactions from GABAergic amacrine cells to RGCs are shown in the upper graph, and the Müller glia to RGCs interactions are shown in the lower graph.

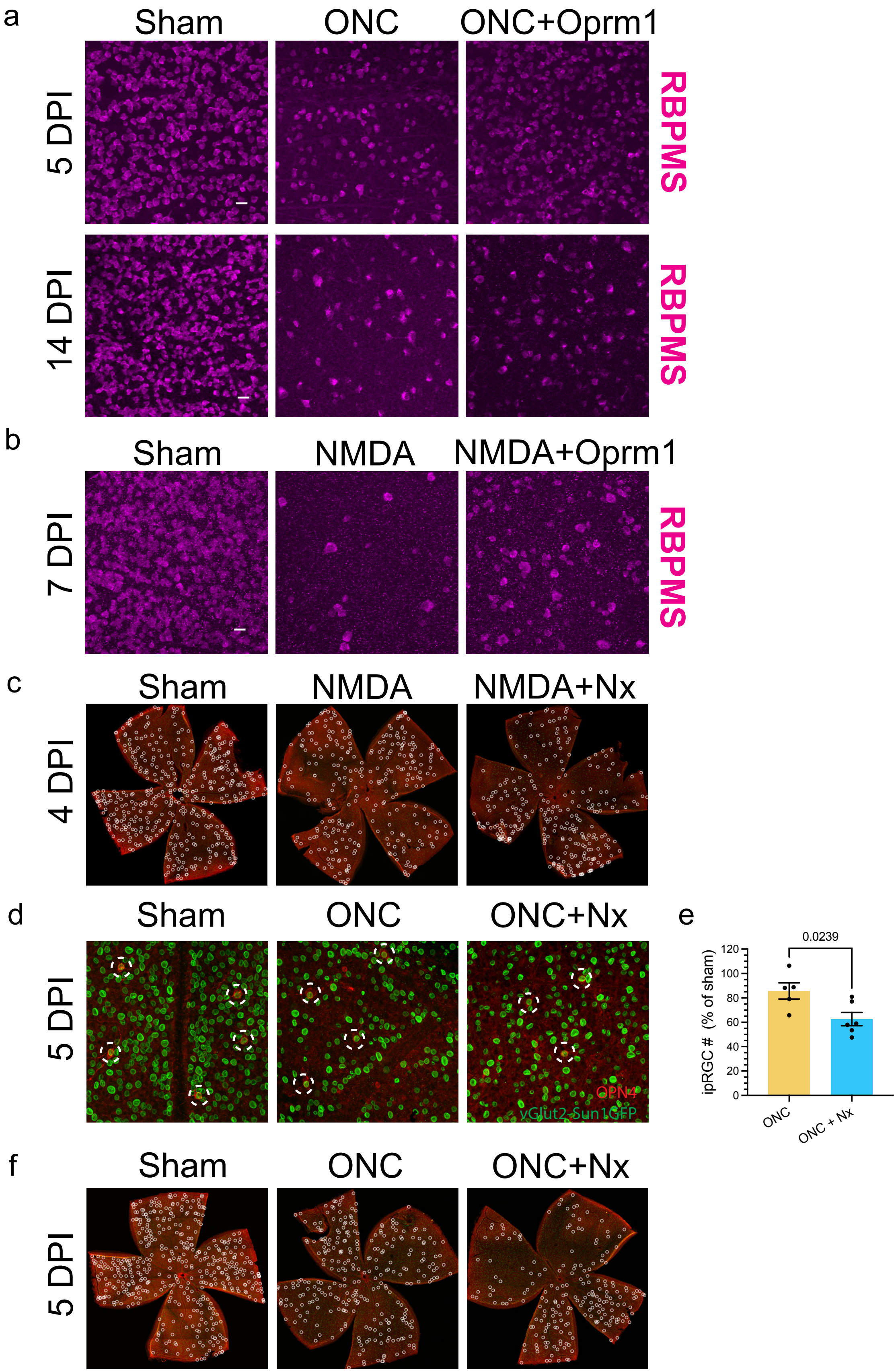

a
Sham
ONC
ONC+Oprm1
5 DPI
14 DPI
RBPMS
RBPMS
b
Sham
NMDA
NMDA+Oprm1
7 DPI
RBPMS
c
Sham
NMDA
NMDA+Nx
4 DPI
d
Sham
ONC
ONC+Nx
5 DPI
OPN4
vGlut2-Sun1GFP
e
0.0239
ipRGC # (% of sham)
0
20
40
60
80
100
120
ONC
ONC + Nx
f
Sham
ONC
ONC+Nx
5 DPI

Fig. S5 Neuroprotective effect of Oprm1 on RGC survival.

a Representative images of retina whole mounts of RBPMS staining 5 days (upper row) or 14 days (lower row) post-ONC. Scale bars: 50 μm, applies to all micrographs. **b** Representative confocal images of retina whole mounts of RBPMS staining seven days following NMDA damage. **c** Representative confocal scans of petal-shaped retina whole mounts showing the OPN4 staining of ipRGC, four days after NMDA damage, compared with co-treatment with NMDA and naloxone injection. White circles label the ipRGCs (OPN4 in red fluorescence). **d** Representative confocal images of retina whole mounts showing ipRGC numbers five days post-ONC. The red channel represents OPN4 staining, and the green fluorescence shows vGlut2-Sun1GFP as a pan-RGC marker. White dashed line circles mark the ipRGCs. **e** Numbers of ipRGC cells survived (OPN4+), as the percentages relative to the sham group. Data are presented as mean ± SEM. ONC group, n=5; ONC+ Nx group, n=6. One-way ANOVA, multiple comparisons. **f** Representative confocal scans of petal-shape retina whole mounts showing ipRGC cell survived five days post-ONC and ONC combined with naloxone injection condition. White circles label the ipRGCs (OPN4 in red fluorescence). Source data are provided as a Source Data file.

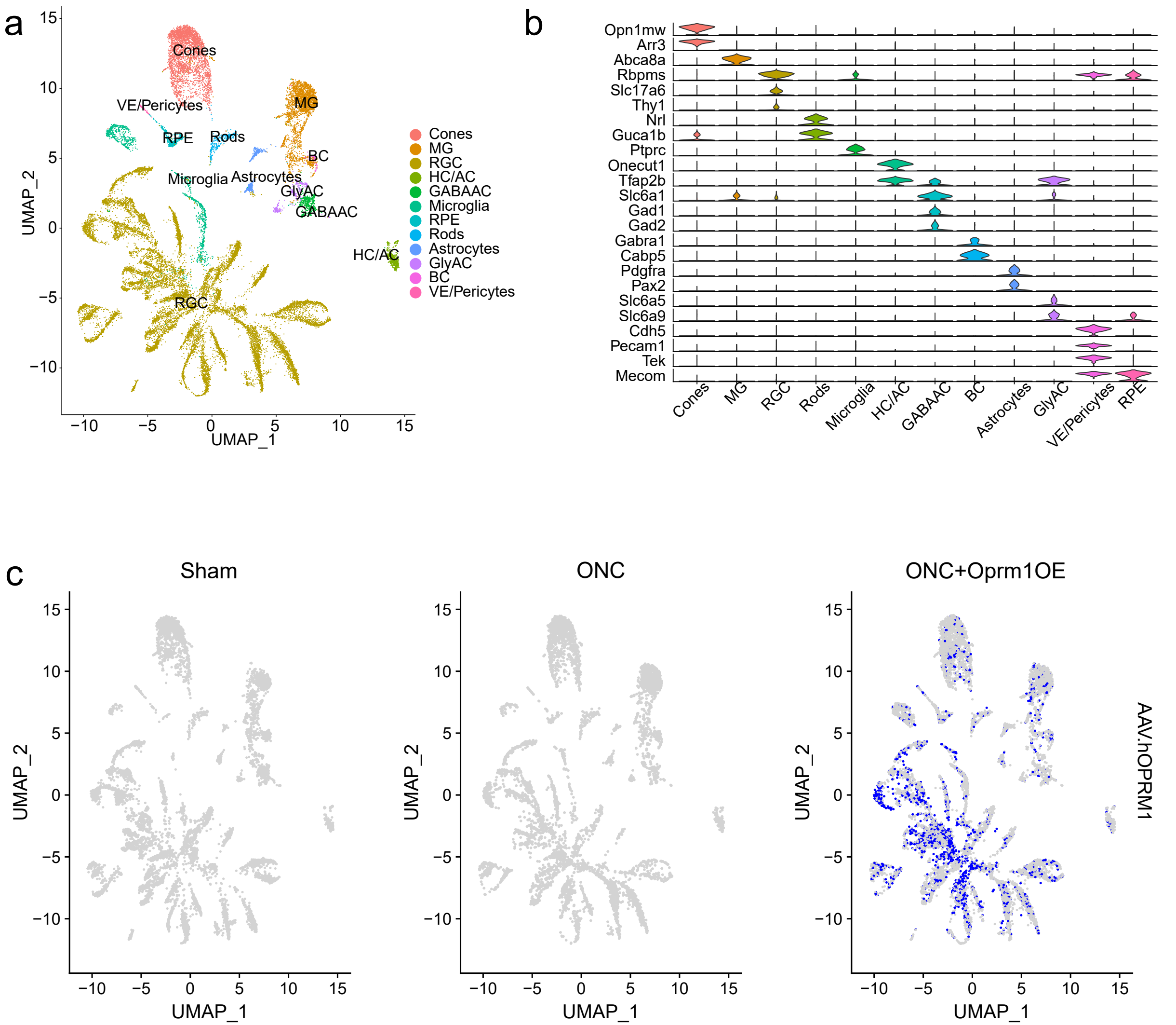
a
Cones
VE/Pericytes
MG
RPE
Rods
BC
Microglia
Astrocytes
GlyAC
GABAAC
HC/AC
RGC
UMAP_1
UMAP_2
Cones
MG
RGC
HC/AC
GABAAC
Microglia
RPE
Rods
Astrocytes
GlyAC
BC
VE/Pericytes
b
Opn1mw
Arr3
Abca8a
Rbpms
Slc17a6
Thy1
Nrl
Guca1b
Ptprc
Onecut1
Tfap2b
Slc6a1
Gad1
Gad2
Gabra1
Cabp5
Pdgfra
Pax2
Slc6a5
Slc6a9
Cdh5
Pecam1
Tek
Mecom
Cones
MG
RGC
Rods
Microglia
HC/AC
GABAAC
BC
Astrocytes
GlyAC
VE/Pericytes
RPE
c
Sham
ONC
ONC+Oprm1OE
UMAP_1
UMAP_2
AAV.hOPRM1

Fig. S6 snRNA-seq analysis on sorted RGC nuclei.

a Retinal cells obtained from the samples. The samples were enriched for RGCs with anti-GFP MACS for vGlut2-Sun1GFP+ pan-RGCs. **b** Expression level of representative known marker genes in retinal cell types. **c** Expression patterns of ectopic human Oprm1 (based on AAV2 WPRE detection) in retinal cells. The ectopic Oprm1 is mainly detected in RGCs.

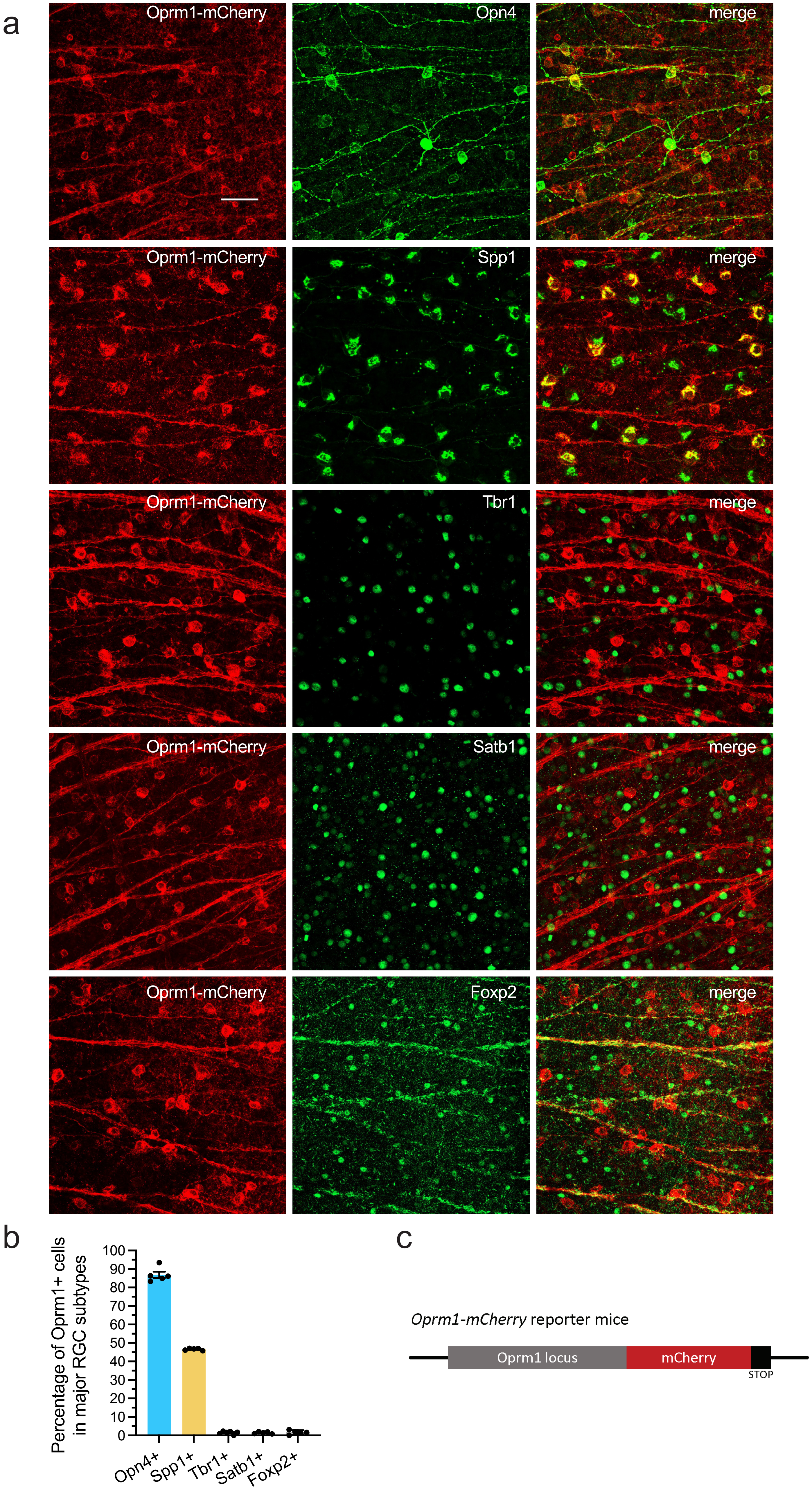
a
Oprm1-mCherry
Opn4
merge
Oprm1-mCherry
Spp1
merge
Oprm1-mCherry
Tbr1
merge
Oprm1-mCherry
Satb1
merge
Oprm1-mCherry
Foxp2
merge
b
Percentage of Oprm1+ cells in major RGC subtypes
0
10
20
30
40
50
60
70
80
90
100
Opn4+
Spp1+
Tbr1+
Satb1+
Foxp2+
c
Oprm1-mCherry reporter mice
Oprm1 locus
mCherry
STOP

Fig. S7 Representative confocal images of whole-mount retinas

a The images show the physiological expression pattern of Oprm1 in multiple RGC subtypes labeled by marker proteins. The red fluorescent is immuno-staining of the mCherry tag in the Oprm1-mCherry mouse strain. The green color stands for immuno-staining of Opn4 (ip-RGC marker), Spp1 (αRGC marker), Tbr1 (T-RGC marker), Satb1 (DS-RGC marker) and Foxp2 (F-RGC marker), respectively. Scale bars: 50 μm. **b** Quantification of the percentage coverage of the Oprm1 expressing cells in Opn4+, Spp1+, Tbr1+, Satb1+, or Foxp2+ major RGC subsets, respectively. **c** The schematic diagram shows the knock-in location of the mCherry tag in the Oprm1-mCherry reporter mouse strain. Source data are provided as a Source Data file.

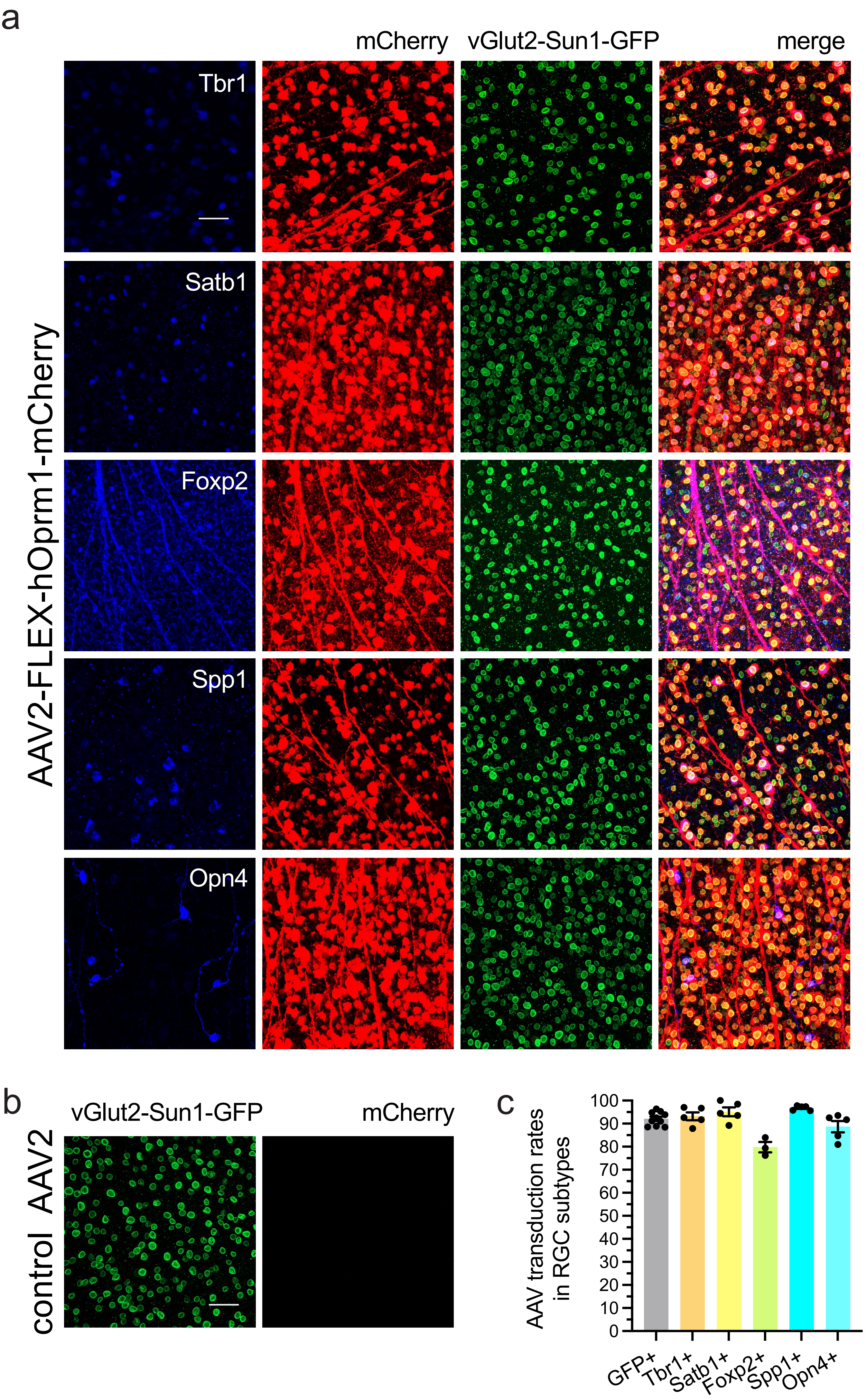
a
mCherry
vGlut2-Sun1-GFP
merge
AAV2-FLEX-hOprm1-mCherry
Tbr1
Satb1
Foxp2
Spp1
Opn4
b
vGlut2-Sun1-GFP
mCherry
control AAV2
c
AAV transduction rates
in RGC subtypes
100
90
80
70
60
50
40
30
20
10
0
GFP+
Tbr1+
Satb1+
Foxp2+
Spp1+
Opn4+

Fig. S8 Representative confocal images of whole-mount retinas with overexpression of Oprm1

a The images show the viral transduction coverage of AAV2-FLEX-hOprm1-mCherry transducing the vGlut2-IRES-Cre; LSL-Sun1-GFP mouse. The blue color stands for immuno-staining of Opn4 (ip-RGC marker), Spp1 (αRGC marker), Tbr1 (T-RGC marker), Satb1 (DS-RGC marker) and Foxp2 (F-RGC marker), respectively. The red fluorescence is the immuno-staining of the mCherry tag expressed by AAV2-FLEX-hOprm1-mCherry. The green fluorescence is GFP immuno-staining of Sun1-GFP to visualize pan-RGCs. Scale bars: 50 μm. **b** Control AAV2 (AAV2-FLEX-Sun1GFP) infected vGlut2-IRES-Cre; LSL-Sun1-GFP mouse retinas did not show signal in anti-mCherry immunostaining. Scale bars: 50 μm. **c** Quantification of viral transduction percent coverage of AAV2-FLEX-hOprm1-mCherry onto pan-RGC marker (Sun1-GFP) or other major RGC subtype markers shown in panel **a**. Source data are provided as a Source Data file.

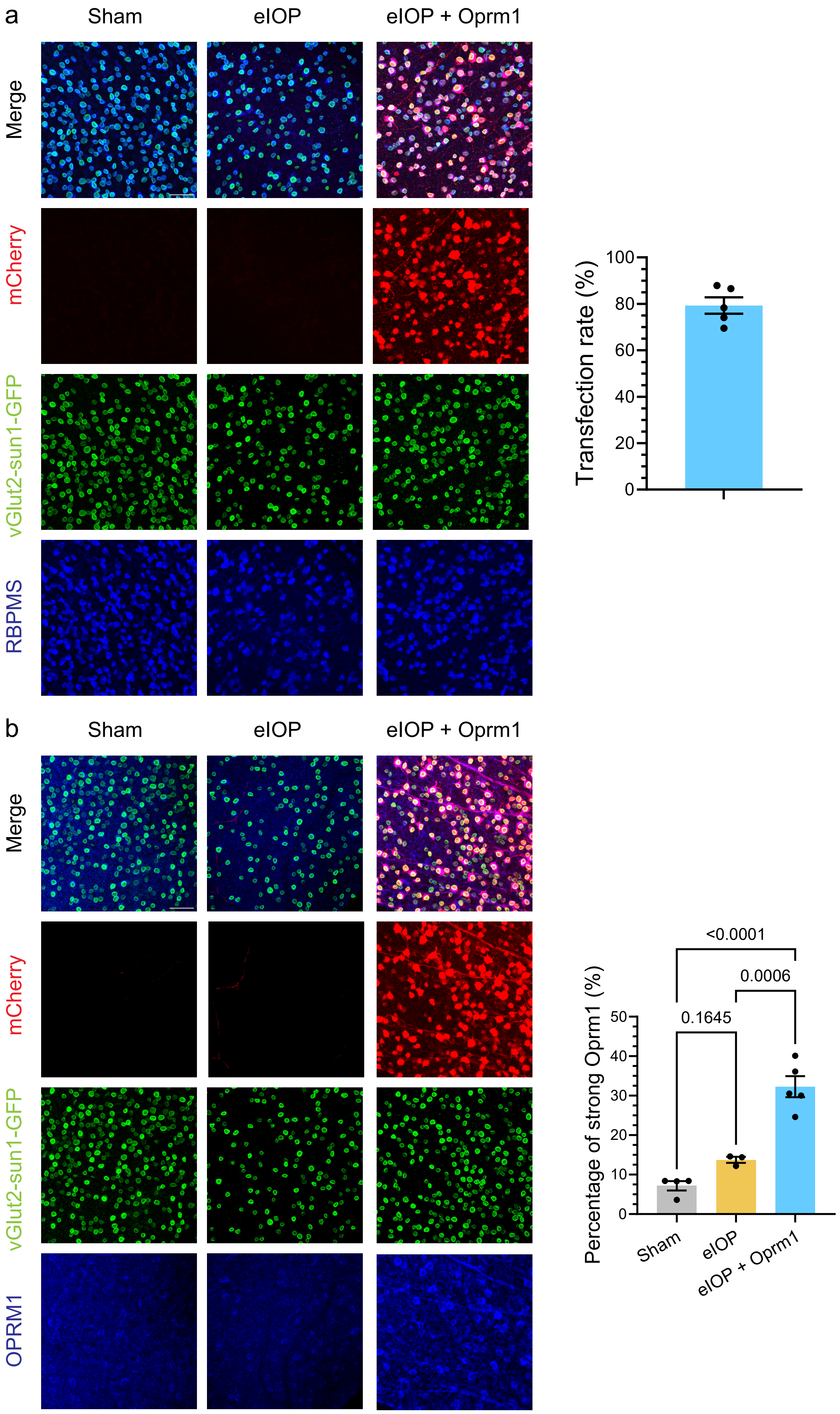
a
Sham
eIOP
eIOP + Oprm1
Merge
mCherry
vGlut2-sun1-GFP
RBPMS
Transfection rate (%)
0
20
40
60
80
100
b
Sham
eIOP
eIOP + Oprm1
Merge
mCherry
vGlut2-sun1-GFP
OPRM1
Percentage of strong Oprm1 (%)
0
10
20
30
40
50
<0.0001
0.0006
0.1645
Sham
eIOP
eIOP + Oprm1

Fig. S9 AAV-mediated *Oprm1* gene transduction in eIOP glaucoma model.
a Representative confocal images of retina whole mounts showing the expression of mCherry in Oprm1 + eIOP group, which was transduced with AAV2-FLEX-mCherry-Oprm1 on vGlut2-Cre; LSL-Sun1GFP mice. The red fluorescent staining is mCherry, the green fluorescence is Sun1GFP in pan-RGCs, and the blue channel represents RBPMS staining. **b** Representative confocal fluorescent images of retina whole mounts showing the expression of mCherry in the Oprm1 + eIOP group, which was transduced with AAV2-FLEX-mCherry-Oprm1, on vGlut2-Cre; LSL-Sun1GFP mice. The red fluorescent staining is mCherry, the green fluorescence is Sun1GFP in pan-RGCs, and the blue channel represents Oprm1 staining. Scale bars: 50 μm. Source data are provided as a Source Data file.

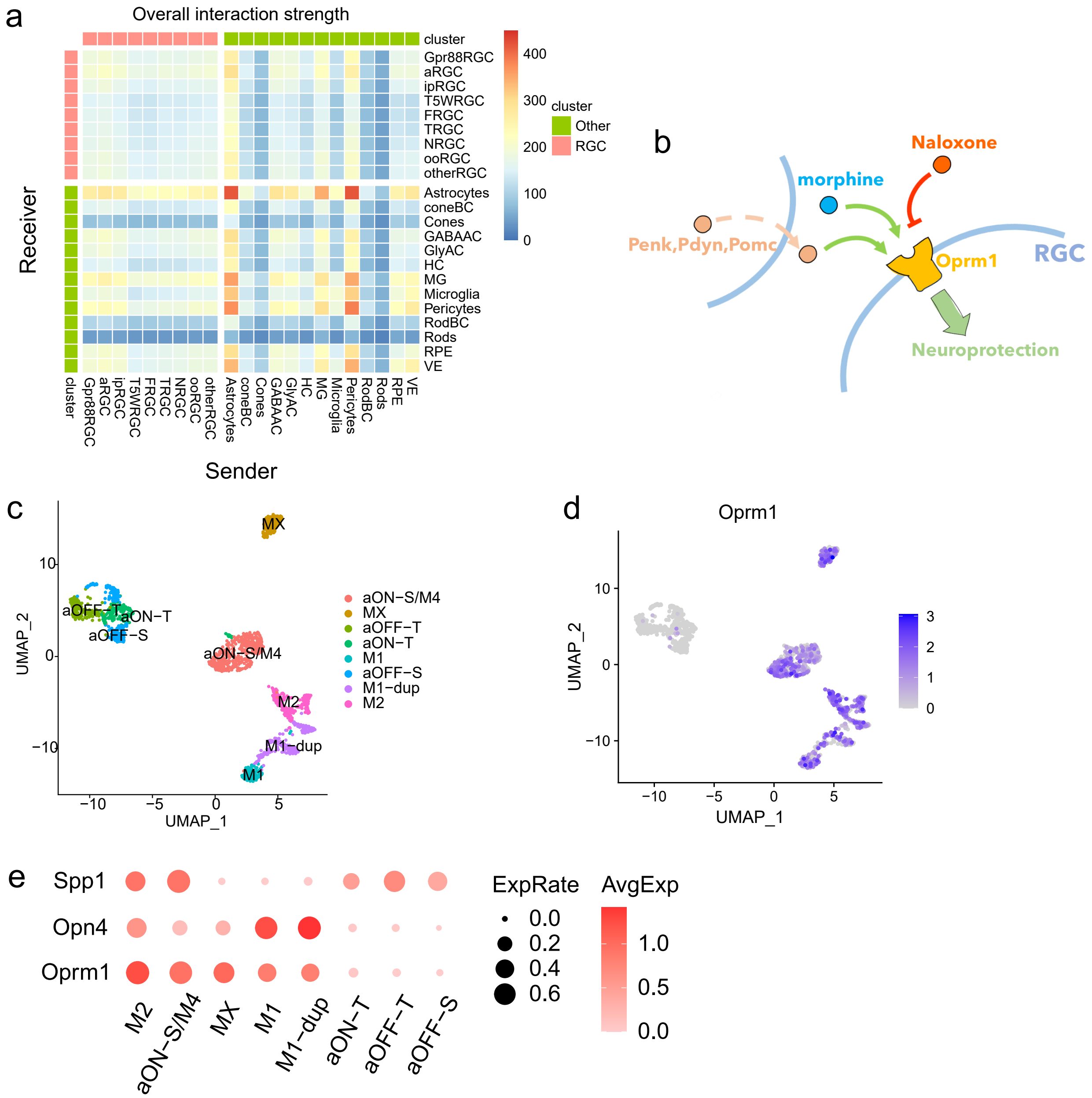

a
Overall interaction strength
cluster
Gpr88RGC
aRGC
ipRGC
T5WRGC
FRGC
TRGC
NRGC
ooRGC
otherRGC
Astrocytes
coneBC
Cones
GABAAC
GlyAC
HC
MG
Microglia
Pericytes
RodBC
Rods
RPE
VE
400
300
200
100
0
cluster
Other
RGC
Receiver
Sender
b
Naloxone
morphine
Penk,Pdyn,Pomc
Oprm1
RGC
Neuroprotection
c
UMAP_2
UMAP_1
MX
aOFF−T
aON−T
aOFF−S
aON−S/M4
M2
M1−dup
M1
aON−S/M4
MX
aOFF−T
aON−T
M1
aOFF−S
M1−dup
M2
d
Oprm1
UMAP_2
UMAP_1
3
2
1
0
e
Spp1
Opn4
Oprm1
M2
aON−S/M4
MX
M1
M1−dup
aON−T
aOFF−T
aOFF−S
ExpRate
0.0
0.2
0.4
0.6
AvgExp
1.0
0.5
0.0

Fig. S10. Topics for discussion.
a Cell-cell interactions between retinal cell types, including those between non-RGC cell types. **b** A schematic for Oprm1 interactions. **c-e** Oprm1 expression in ipRGC and αRGC subtypes. **c** clustering and annotation of ipRGCs and αRGCs. **d** UMAP of the expression level of Oprm1. **e** Expression level of Opn4, Spp1, and Oprm1 in subtypes.
