# Supplementary material for: Intercellular communication atlas reveals Oprm1 as a neuroprotective factor for retinal ganglion cells
Source: Nat Commun. 2024 Mar 11;15:2206. doi: 10.1038/s41467-024-46428-z (PMC11636819; doi:10.1038/s41467-024-46428-z)
Supplement: Supplementary file 3 — Reporting Summary [file 41467_2024_46428_MOESM3_ESM.pdf]

nature portfolio

Corresponding author(s): Jiang Qian

Last updated by author(s): Feb 16, 2024

Reporting Summary

Nature Portfolio wishes to improve the reproducibility of the work that we publish. This form provides structure for consistency and transparency in reporting. For further information on Nature Portfolio policies, see our Editorial Policies and the Editorial Policy Checklist.

Statistics

For all statistical analyses, confirm that the following items are present in the figure legend, table legend, main text, or Methods section.

n/a	Confirmed	
☐	☒	The exact sample size (*n*) for each experimental group/condition, given as a discrete number and unit of measurement
☐	☒	A statement on whether measurements were taken from distinct samples or whether the same sample was measured repeatedly
☐	☒	The statistical test(s) used AND whether they are one- or two-sided<br>*Only common tests should be described solely by name; describe more complex techniques in the Methods section.*
☐	☒	A description of all covariates tested
☐	☒	A description of any assumptions or corrections, such as tests of normality and adjustment for multiple comparisons
☐	☒	A full description of the statistical parameters including central tendency (e.g. means) or other basic estimates (e.g. regression coefficient) AND variation (e.g. standard deviation) or associated estimates of uncertainty (e.g. confidence intervals)
☐	☒	For null hypothesis testing, the test statistic (e.g. *F*, *t*, *r*) with confidence intervals, effect sizes, degrees of freedom and *P* value noted<br>*Give P values as exact values whenever suitable.*
☒	☐	For Bayesian analysis, information on the choice of priors and Markov chain Monte Carlo settings
☒	☐	For hierarchical and complex designs, identification of the appropriate level for tests and full reporting of outcomes
☐	☒	Estimates of effect sizes (e.g. Cohen's *d*, Pearson's *r*), indicating how they were calculated

Our web collection on statistics for biologists contains articles on many of the points above.

Software and code

Policy information about availability of computer code

Data collection	The scRNA-seq libraries were prepared using the 10X Genomics Chromium 3' Single-Cell Gene Expression V3 Kit. The snRNA-seq were prepared using 10X Genomics Single-Nuclei 3' HT platform. Libraries were sequenced with the NovaSeq S2 100 platform. FASTQ files were shared from the Johns Hopkins Single Cell and Transcriptomics Core.
Data analysis	Raw reads of adult mouse retina scRNA-seq data before and post-ONC (control, 12h, 1d, and 2d) were mapped to mm10 and expression matrices were generated using Cell Ranger 3.0.2 from 10X Genomics. We created a Seurat object for each sample using Seurat v4.0.5. For the whole retina dataset, cells were removed if their nUMI was <800 or >30000, nGene was <350 or >7500, mitochondrial gene rate was >20%, or log10GenesPerUMI was <0.8. For the RGC dataset, we removed cells with nUMI <500, nGene <250, mitochondrial rate >20%, or log10GenesPerUMI <0.8. We then used Scrublet v0.2.3 to remove predicted doublets with default parameters for both data sets. Furthermore, genes detected in less than 5 cells in each sample were also removed from the data. As a result, 56,531 cells from the whole retina dataset and 86,426 cells from the RGC dataset were obtained for downstream analysis. For each dataset, Seurat objects of the samples were then normalized, integrated, clustered, and visualized through UMAP using Seurat v4.0.5.<br><br>For enriched RGC snRNA-seq data from sham condition, post-ONC (5d), and post-ONC (5d) with hOprm1 overexpression samples, raw reads were mapped to mm10, and expression matrices were generated using Cell Ranger 7.0.0 from 10x Genomics. A Seurat object for each sample was created using Seurat v4.3.0. For sham data, cells were removed if nUMI was < 1000 or > 50000, nGene was < 500 or > 10000, mitochondrial rate was > 10%, or log10GenesPerUMI was < 0.8. For ONC and ONC with Oprm1 overexpression data, cells were removed if nUMI was < 1000 or > 30000, nGene was < 500 or > 7000, mitochondrial rate was > 10%, or log10GenesPerUMI was < 0.8. We then removed predicted doublets using DoubletFinder v2.0.3 with its standard pipeline for each sample (McGinnis et al., 2019). In total, 26170 cells were used for downstream analysis. Specifically, 9306, 6060, and 10804 cells were obtained from the sham, ONC, and ONC with Oprm1 overexpression samples, respectively. The filtered Seurat objects were then normalized, integrated, clustered, and visualized through UMAP

using Seurat v4.3.0. After cell type annotation, 15803 RGCs were obtained, with 6348, 3314, and 6141 RGCs from the sham, ONC, and ONC with Oprm1 overexpression samples, respectively. We then took the subset of the Seurat objects of each sample by removing non-RGCs and performed normalization, integration, clustering, and UMAP visualization again using Seurat.

For ligand-receptor interaction analysis, we used LRLoop software (https://github.com/Pinlyu3/LRLoop)

For manuscripts utilizing custom algorithms or software that are central to the research but not yet described in published literature, software must be made available to editors and reviewers. We strongly encourage code deposition in a community repository (e.g. GitHub). See the Nature Portfolio guidelines for submitting code & software for further information.

Data

Policy information about availability of data

All manuscripts must include a data availability statement. This statement should provide the following information, where applicable:

- Accession codes, unique identifiers, or web links for publicly available datasets
- A description of any restrictions on data availability
- For clinical datasets or third party data, please ensure that the statement adheres to our policy

The scRNA-seq and snRNA-seq data generated in this study have been deposited in the GEO database under accession code GSE241268 [https://www.ncbi.nlm.nih.gov/geo/query/acc.cgi?acc=GSE241268], GSE248537 [https://www.ncbi.nlm.nih.gov/geo/query/acc.cgi], and GSE248868 [https://www.ncbi.nlm.nih.gov/geo/query/acc.cgi]. The scRNA-seq data generated in this study can also be visualized at Broad Institute Single Cell portal under accession code SCP2423 [https://singlecell.broadinstitute.org/single_cell/study/SCP2423/single-cell-rna-seq-of-adult-mouse-whole-retinal-cell-types-upon-optic-nerve-crush-injury]. Source data are provided with this paper.

Research involving human participants, their data, or biological material

Policy information about studies with human participants or human data. See also policy information about sex, gender (identity/presentation), and sexual orientation and race, ethnicity and racism.

Reporting on sex and gender	N/A
Reporting on race, ethnicity, or other socially relevant groupings	N/A
Population characteristics	N/A
Recruitment	N/A
Ethics oversight	N/A

Note that full information on the approval of the study protocol must also be provided in the manuscript.

Field-specific reporting

Please select the one below that is the best fit for your research. If you are not sure, read the appropriate sections before making your selection.

☒ Life sciences ☐ Behavioural & social sciences ☐ Ecological, evolutionary & environmental sciences

For a reference copy of the document with all sections, see nature.com/documents/nr-reporting-summary-flat.pdf

Life sciences study design

All studies must disclose on these points even when the disclosure is negative.

Sample size	sample sizes were selected according to previous publications [PMID: 31784286; PMID: 34297923; PMID: 28641113] in similar research fields, also based on pioneering pre-experiments.
Data exclusions	As described in details in the Method section related to the quality control of scRNA-seq and snRNA-seq data, computational filtering of low-quality cells or doublet, etc. were performed with standard unbiased workflow.
Replication	The biological replicates were described in the figure legends or the Method section and Figure Legends.
Randomization	The age and sex of mice were randomized for in vivo experiments.
Blinding	The investigators responsible for data analysis were blinded to group allocation during data collection. The whole-genome sequencing is an unbiased method for data acquisition. The computational analysis and predictions were conducted based on integration, and were without possible bias toward specific group(s). Experimenter performing imaging analysis were blinded from the treatment condition names.

Reporting for specific materials, systems and methods

We require information from authors about some types of materials, experimental systems and methods used in many studies. Here, indicate whether each material, system or method listed is relevant to your study. If you are not sure if a list item applies to your research, read the appropriate section before selecting a response.

Materials & experimental systems

n/a	Involved in the study
☐	☒ Antibodies
☒	☐ Eukaryotic cell lines
☒	☐ Palaeontology and archaeology
☐	☒ Animals and other organisms
☒	☐ Clinical data
☒	☐ Dual use research of concern
☒	☐ Plants

Methods

n/a	Involved in the study
☒	☐ ChIP-seq
☒	☐ Flow cytometry
☒	☐ MRI-based neuroimaging

Antibodies

Antibodies used

The following primary antibodies were used accordingly: rabbit polyclonal anti-RBPMS (Invitrogen, PA5-31231, 1:500), chicken polyclonal anti-GFP (Invitrogen, A10262, 1:500), rabbit polyclonal anti-OPRM1 (Invitrogen, PA1-24628, 1:500), rabbit polyclonal anti-Opn4 (Invitrogen, PA1-780, 1:500) for Suppl. Fig. 9, rabbit polyclonal anti-Melanopsin (AB-N38, Advanced Targeting Systems, 1:500), rabbit mAb anti-Tbr1(CST #49661, 1:200), rabbit mAb anti-Satb1 (abcam ab109122, 1:200), rabbit polyclonal anti-Foxp2 (abcam ab16046, 1:500), goat polyclonal IgG anti-Osteopontin (biotechne AF808, diluted to 5ug/mL). For staining the mCherry tag of the MOR-mCherry reporter mice (JAX #029013) or the AAV2-FLEX-hOprm1-mCherry virus-infected samples, a rat mAb anti-mCherry was used (Invitrogen, M11217, 1:500). Following secondary antibodies were used correspondingly: Alexa Fluor 647 goat anti-rabbit IgG (Invitrogen A21245, 1:500), Alexa Fluor 488 goat anti-chicken IgY (Invitrogen A11039, 1:500), Alexa Fluor 568 goat anti-rabbit IgG (Invitrogen A11011, 1:500), Alexa Fluor 568 goat anti-rat IgG (Invitrogen A11077, 1:500), donkey anti-Goat IgG Alexa Fluor Plus 647 (Thermo A32849TR, 1:500), donkey anti-Rat Alexa Fluor 594 (Thermo A21209, 1:500), donkey anti-Rabbit IgG Alexa Fluor 488 (Thermo A21206, 1:500), donkey anti-Chicken IgY Alexa Fluor 488 (Thermo A78948, 1:500).

Validation

N/A

Animals and other research organisms

Policy information about studies involving animals; ARRIVE guidelines recommended for reporting animal research, and Sex and Gender in Research

Laboratory animals

All animal experiments were approved by the Institutional Animal Care and Use Committees (IACUC) at Johns Hopkins University School of Medicine. The mice were maintained in a climate and light/dark cycle (14h/10h) controlled pathogen-free facility, with continuous access to food and water. All studies were conducted on adult mice aged 2 to 3 months. Both male and female mice were included in the study. The following mouse strains were utilized: C57BL/6 mice (JAX #000664), vGlut2-IRES-Cre (JAX #016963), CAG-LSL-Sun1/sfGFP mice (JAX #021039), and MOR-mCherry reporter mice (JAX #029013), which were all available at the Jackson Laboratory (Bar Harbor, ME). The homozygotes vGlut2-Cre; LSL-Sun1GFP mice were generated by breeding and backcross.

Wild animals

N/A

Reporting on sex

both male and female mice were used.

Field-collected samples

N/A

Ethics oversight

All animal experiments were approved by the Institutional Animal Care and Use Committees (IACUC) at Johns Hopkins University School of Medicine.

Note that full information on the approval of the study protocol must also be provided in the manuscript.

Plants

Seed stocks	NA
Novel plant genotypes	NA
Authentication	NA
